# (*E*)-1-(5-Iodo­thio­phen-2-yl)-3-(3,4,5-trimeth­oxy­phen­yl)prop-2-en-1-one

**DOI:** 10.1107/S1600536812038226

**Published:** 2012-09-12

**Authors:** Vijayan Viswanathan, Thothadri Srinivasan, Ayyavu Thirunarayanan, Perumal Rajakumar, Devadasan Velmurugan

**Affiliations:** a Centre of Advanced Study in Crystallography and Biophysics, University of Madras, Guindy Campus, Chennai 600 025, India; b Department of Organic Chemistry, University of Madras, Guindy Campus, Chennai 600 025, India

## Abstract

In the title compound, C_16_H_15_IO_4_S, the dihedral angle between the thio­phene and benzene rings is 11.50 (2)°. The methoxy O atoms deviate by 0.0060 (2), −0.1319 (2) and 0.0426 (2) Å from the phenyl ring plane. The crystal packing features C—H⋯O hydrogen bonds, which link the molecules into *C*(11) chains propagating in [100xxx].

## Related literature

For the biological activity of chalcones, see: Di Carlo *et al.* (1999 ▶); Lin *et al.* (2002 ▶). For a related structure, see Ranjith *et al.* (2010 ▶).
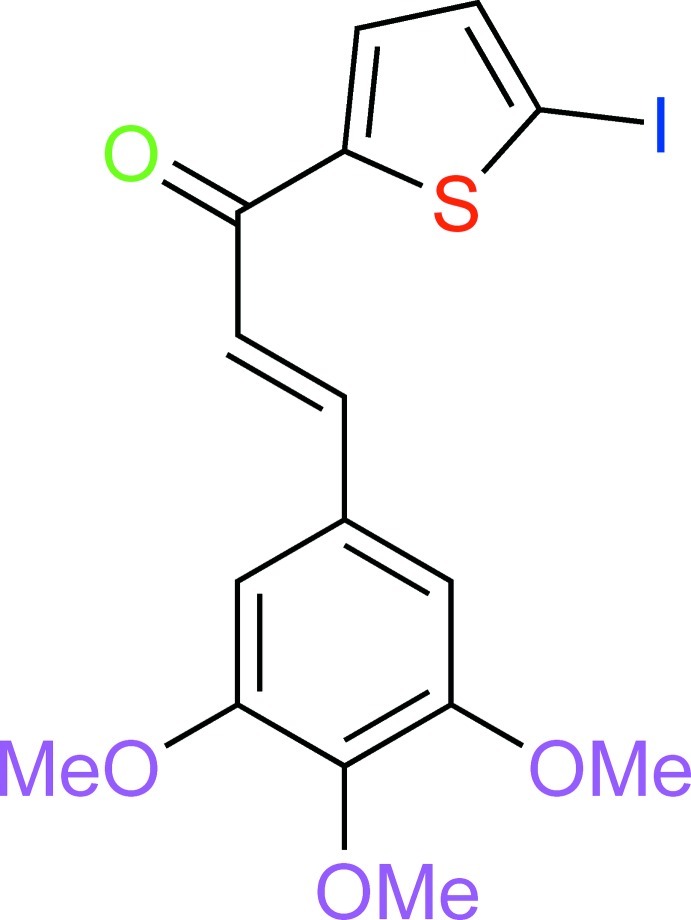


## Experimental

### 

#### Crystal data

C_16_H_15_IO_4_S
*M* *_r_* = 430.25
Orthorhombic, 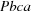

*a* = 17.2328 (12) Å
*b* = 8.1885 (6) Å
*c* = 23.7049 (17) Å
*V* = 3345.0 (4) Å^3^
*Z* = 8
Mo *K*α radiation
μ = 2.05 mm^−1^
*T* = 293 K
0.30 × 0.25 × 0.20 mm

#### Data collection

Bruker APEXII area-detector diffractometer
Absorption correction: multi-scan (*SADABS*; Bruker, 2008 ▶) *T*_min_ = 0.578, *T*_max_ = 0.684
17211 measured reflections
4092 independent reflections
3263 reflections with *I* > 2σ(*I*)
*R*_int_ = 0.020

#### Refinement

*R*[*F* ^2^ > 2σ(*F* ^2^)] = 0.025
*wR*(*F* ^2^) = 0.062
*S* = 1.06
4092 reflections
202 parameters
H-atom parameters constrained
Δρ_max_ = 0.45 e Å^−3^
Δρ_min_ = −0.51 e Å^−3^

### 

Data collection: *APEX2* (Bruker, 2008 ▶); cell refinement: *SAINT* (Bruker, 2008 ▶); data reduction: *SAINT*; program(s) used to solve structure: *SHELXS97* (Sheldrick, 2008 ▶); program(s) used to refine structure: *SHELXL97* (Sheldrick, 2008 ▶); molecular graphics: *ORTEP* 3 (Farrugia, 1997 ▶); software used to prepare material for publication: *SHELXL97* and *PLATON* (Spek, 2009 ▶).

## Supplementary Material

Crystal structure: contains datablock(s) global, I. DOI: 10.1107/S1600536812038226/bt6834sup1.cif

Structure factors: contains datablock(s) I. DOI: 10.1107/S1600536812038226/bt6834Isup2.hkl

Supplementary material file. DOI: 10.1107/S1600536812038226/bt6834Isup3.cml

Additional supplementary materials:  crystallographic information; 3D view; checkCIF report

## Figures and Tables

**Table 1:** Hydrogen-bond geometry (Å, °)

*D*—H⋯*A*	*D*—H	H⋯*A*	*D*⋯*A*	*D*—H⋯*A*
C15—H15⋯O2^i^	0.93	2.45	3.341 (3)	159

Symmetry code: (i) 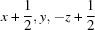.

**Fig. 1.**
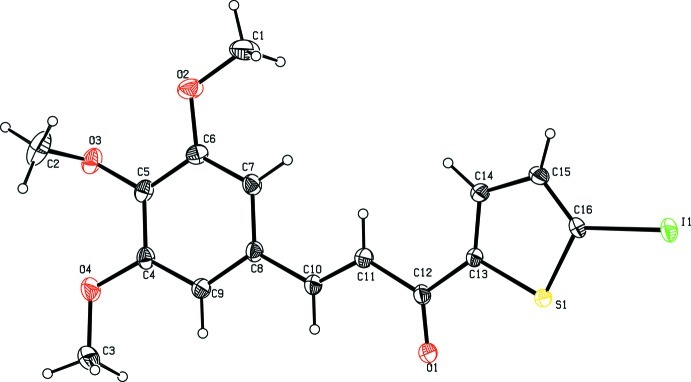
The molecular structure of the title compound, showing displacement ellipsoids drawn at the 30% probability level. H atoms are presented as small spheres of arbitrary radii.

**Fig. 2.**
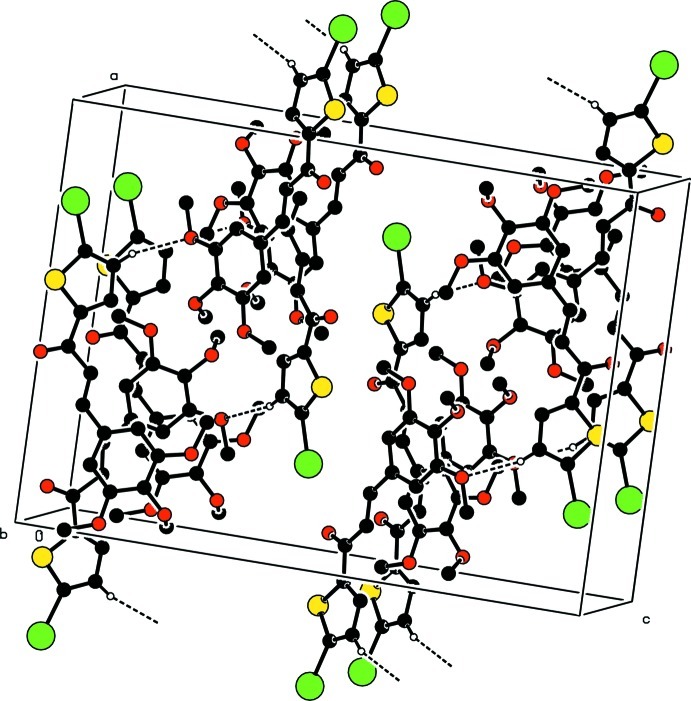
The crystal packing of the title compound viewed down the a axis. H-atoms not involved in H-bonds have been excluded for clarity.

## supplementary crystallographic information

### Comment

Chalcones are one of the major classes of natural products with widespread distribution in fruits, vegetables, spices, tea and soy based foodstuff and have recently been the subject of great interest because of their interesting pharmacological activities (Di Carlo *et al.*, 1999). Chalcones and flavonoids as anti-tuberculosis agents are also reported (Lin *et al.*, 2002). Against this background and in order to obtain detailed information on molecular conformations in the solid state, an X-ray study of the title compound was carried out.

In the title compound, the dihedral angle between the thiophene ring (S1/C13/C14/C15/C16) and the three methoxy substituted phenyl ring is 11.50 (2)°. The oxygen atoms O2, O3 & O4 attached with the phenyl ring (C4/C5/C6/C7/C8/C9) deviate by 0.0060 (2)Å, -0.1319 (2)Å and 0.0426 (2)Å, respectively. The iodine atom (I1) attached with the thiophene ring deviates by a value of -0.0195 (1)Å. The crystal packing is stabilized by intermolecular C–H···O hydrogen bonds.

### Experimental

The iodo chalcone was prepared by condensation of 2-acetyl,5-iodo-thiophene (1 equiv) with 3,4,5-trimethoxybenzaldehyde (1 equiv) with 10% NaOH solution (10 mL) in ethyl alcohol (100 mL) stirred at room temperature for 12 h. The reaction mixture was poured into ice water (100 ml) and acidified with dilute HCl. The precipitated product was filtered and washed many times with water and dried to give the crude product. This was recrystallised in methyl alcohol to afford the pure iodo chalcone in dark brown colour. Yield: 82%

### Refinement

Hydrogen atoms were placed in calculated positions with C_aromatic_—H = 0.93 Å and C_methyl_—H = 0.96Å and refined using a riding model with *U*_iso_(H) = 1.2 *U*_eq_(C) or *U*_iso_(H) = 1.5 *U*_eq_(C_methyl_). The methyl groups were allowed to rotate but not to tip.

### Crystal data

**Table:**

C_16_H_15_IO_4_S	*F*(000) = 1696
*M**_r_* = 430.25	*D*_x_ = 1.709 Mg m^−^^3^
Orthorhombic, *P**b**c**a*	Mo *K*α radiation, λ = 0.71073 Å
Hall symbol: -P 2ac 2ab	Cell parameters from 4092 reflections
*a* = 17.2328 (12) Å	θ = 1.7–28.2°
*b* = 8.1885 (6) Å	µ = 2.05 mm^−^^1^
*c* = 23.7049 (17) Å	*T* = 293 K
*V* = 3345.0 (4) Å^3^	Block, colourless
*Z* = 8	0.30 × 0.25 × 0.20 mm

### Data collection

**Table:**

Bruker APEXII area-detector diffractometer	4092 independent reflections
Radiation source: fine-focus sealed tube	3263 reflections with *I* > 2σ(*I*)
Graphite monochromator	*R*_int_ = 0.020
ω and φ scans	θ_max_ = 28.2°, θ_min_ = 1.7°
Absorption correction: multi-scan (*SADABS*; Bruker, 2008)	*h* = −22→12
*T*_min_ = 0.578, *T*_max_ = 0.684	*k* = −8→10
17211 measured reflections	*l* = −25→31

### Refinement

**Table:**

Refinement on *F*^2^	Primary atom site location: structure-invariant direct methods
Least-squares matrix: full	Secondary atom site location: difference Fourier map
*R*[*F*^2^ > 2σ(*F*^2^)] = 0.025	Hydrogen site location: inferred from neighbouring sites
*wR*(*F*^2^) = 0.062	H-atom parameters constrained
*S* = 1.06	*w* = 1/[σ^2^(*F*_o_^2^) + (0.0255*P*)^2^ + 1.7451*P*] where *P* = (*F*_o_^2^ + 2*F*_c_^2^)/3
4092 reflections	(Δ/σ)_max_ = 0.003
202 parameters	Δρ_max_ = 0.45 e Å^−^^3^
0 restraints	Δρ_min_ = −0.51 e Å^−^^3^

### Special details

**Table:**

Geometry. All esds (except the esd in the dihedral angle between two l.s. planes) are estimated using the full covariance matrix. The cell esds are taken into account individually in the estimation of esds in distances, angles and torsion angles; correlations between esds in cell parameters are only used when they are defined by crystal symmetry. An approximate (isotropic) treatment of cell esds is used for estimating esds involving l.s. planes.
Refinement. Refinement of F^2^ against ALL reflections. The weighted R-factor wR and goodness of fit S are based on F^2^, conventional R-factors R are based on F, with F set to zero for negative F^2^. The threshold expression of F^2^ > 2sigma(F^2^) is used only for calculating R-factors(gt) etc. and is not relevant to the choice of reflections for refinement. R-factors based on F^2^ are statistically about twice as large as those based on F, and R- factors based on ALL data will be even larger.

### Fractional atomic coordinates and isotropic or equivalent isotropic displacement parameters (Å^2^)

**Table:**

	*x*	*y*	*z*	*U*_iso_*/*U*_eq_	
I1	0.772746 (8)	0.12196 (2)	0.038840 (8)	0.04680 (7)	
S1	0.58646 (3)	0.18751 (8)	0.02816 (2)	0.04050 (13)	
O3	0.06960 (10)	−0.0979 (2)	0.24845 (7)	0.0542 (5)	
O2	0.21744 (11)	−0.1501 (3)	0.27590 (8)	0.0596 (5)	
O1	0.41760 (9)	0.2434 (2)	0.02460 (7)	0.0520 (4)	
O4	0.02969 (9)	0.0186 (3)	0.14688 (8)	0.0567 (5)	
C8	0.24287 (13)	0.0457 (3)	0.13714 (9)	0.0390 (5)	
C13	0.51699 (12)	0.1111 (3)	0.07348 (9)	0.0351 (5)	
C5	0.12645 (14)	−0.0588 (3)	0.21007 (9)	0.0409 (5)	
C4	0.10723 (13)	0.0096 (3)	0.15811 (10)	0.0407 (5)	
C10	0.30318 (13)	0.1074 (3)	0.09885 (10)	0.0412 (5)	
H10	0.2866	0.1721	0.0690	0.049*	
C7	0.26207 (13)	−0.0265 (3)	0.18831 (9)	0.0417 (5)	
H7	0.3139	−0.0397	0.1982	0.050*	
C15	0.63199 (13)	0.0081 (3)	0.10998 (10)	0.0422 (5)	
H15	0.6634	−0.0502	0.1347	0.051*	
C16	0.65890 (12)	0.0932 (3)	0.06498 (10)	0.0363 (5)	
C14	0.55074 (13)	0.0186 (3)	0.11476 (9)	0.0402 (5)	
H14	0.5227	−0.0324	0.1433	0.048*	
C6	0.20423 (15)	−0.0790 (3)	0.22465 (9)	0.0421 (5)	
C9	0.16523 (13)	0.0637 (3)	0.12198 (10)	0.0418 (5)	
H9	0.1523	0.1118	0.0877	0.050*	
C11	0.37838 (13)	0.0804 (3)	0.10249 (10)	0.0425 (5)	
H11	0.3963	0.0124	0.1311	0.051*	
C12	0.43547 (13)	0.1525 (3)	0.06352 (10)	0.0384 (5)	
C3	0.00578 (16)	0.0851 (4)	0.09421 (12)	0.0601 (7)	
H3A	0.0247	0.1949	0.0908	0.090*	
H3B	−0.0499	0.0852	0.0922	0.090*	
H3C	0.0264	0.0199	0.0641	0.090*	
C1	0.2956 (2)	−0.1641 (5)	0.29405 (13)	0.0774 (10)	
H1A	0.3240	−0.2302	0.2678	0.116*	
H1B	0.2970	−0.2141	0.3307	0.116*	
H1C	0.3186	−0.0575	0.2960	0.116*	
C2	0.0342 (2)	−0.2524 (4)	0.24123 (16)	0.0881 (12)	
H2A	0.0099	−0.2572	0.2048	0.132*	
H2B	−0.0041	−0.2684	0.2701	0.132*	
H2C	0.0729	−0.3363	0.2439	0.132*	

### Atomic displacement parameters (Å^2^)

**Table:**

	*U*^11^	*U*^22^	*U*^33^	*U*^12^	*U*^13^	*U*^23^
I1	0.02665 (9)	0.04840 (11)	0.06536 (12)	−0.00074 (6)	0.00204 (7)	−0.00160 (8)
S1	0.0274 (3)	0.0511 (3)	0.0430 (3)	−0.0001 (2)	0.0009 (2)	0.0131 (3)
O3	0.0510 (10)	0.0671 (12)	0.0447 (9)	−0.0104 (9)	0.0219 (8)	−0.0070 (9)
O2	0.0588 (12)	0.0806 (14)	0.0393 (9)	0.0107 (10)	0.0099 (8)	0.0124 (9)
O1	0.0302 (8)	0.0679 (11)	0.0580 (10)	−0.0010 (8)	−0.0014 (7)	0.0209 (9)
O4	0.0311 (9)	0.0854 (14)	0.0537 (10)	−0.0024 (9)	0.0062 (7)	0.0030 (10)
C8	0.0313 (11)	0.0469 (14)	0.0387 (11)	−0.0043 (10)	0.0050 (9)	−0.0018 (11)
C13	0.0301 (10)	0.0406 (12)	0.0347 (10)	−0.0029 (9)	0.0028 (8)	0.0021 (10)
C5	0.0411 (12)	0.0431 (12)	0.0384 (11)	−0.0027 (10)	0.0139 (10)	−0.0068 (10)
C4	0.0324 (11)	0.0482 (13)	0.0413 (12)	−0.0019 (10)	0.0072 (9)	−0.0078 (10)
C10	0.0324 (11)	0.0512 (14)	0.0398 (12)	−0.0052 (10)	0.0022 (9)	0.0034 (11)
C7	0.0333 (12)	0.0531 (15)	0.0388 (11)	0.0007 (10)	0.0037 (9)	−0.0032 (11)
C15	0.0353 (12)	0.0503 (14)	0.0411 (12)	0.0051 (10)	−0.0052 (9)	0.0055 (11)
C16	0.0263 (10)	0.0400 (12)	0.0425 (12)	0.0002 (9)	−0.0035 (9)	−0.0044 (10)
C14	0.0364 (12)	0.0489 (14)	0.0352 (11)	−0.0006 (10)	0.0033 (9)	0.0064 (10)
C6	0.0490 (13)	0.0455 (13)	0.0318 (11)	0.0042 (11)	0.0071 (10)	−0.0035 (10)
C9	0.0369 (12)	0.0500 (13)	0.0384 (12)	−0.0013 (10)	0.0043 (9)	0.0022 (11)
C11	0.0340 (12)	0.0531 (14)	0.0404 (12)	−0.0008 (10)	0.0033 (9)	0.0054 (11)
C12	0.0300 (11)	0.0445 (13)	0.0405 (11)	−0.0030 (9)	0.0000 (9)	−0.0003 (10)
C3	0.0392 (14)	0.0727 (19)	0.0684 (18)	0.0050 (13)	−0.0044 (13)	0.0029 (15)
C1	0.070 (2)	0.111 (3)	0.0507 (16)	0.024 (2)	0.0007 (15)	0.0221 (18)
C2	0.099 (3)	0.076 (2)	0.089 (2)	−0.034 (2)	0.044 (2)	−0.0086 (19)

### Geometric parameters (Å, º)

**Table:**

I1—C16	2.071 (2)	C10—H10	0.9300
S1—C16	1.708 (2)	C7—C6	1.386 (3)
S1—C13	1.726 (2)	C7—H7	0.9300
O3—C5	1.375 (3)	C15—C16	1.356 (3)
O3—C2	1.414 (3)	C15—C14	1.407 (3)
O2—C6	1.366 (3)	C15—H15	0.9300
O2—C1	1.418 (4)	C14—H14	0.9300
O1—C12	1.225 (3)	C9—H9	0.9300
O4—C4	1.364 (3)	C11—C12	1.473 (3)
O4—C3	1.423 (3)	C11—H11	0.9300
C8—C7	1.390 (3)	C3—H3A	0.9600
C8—C9	1.393 (3)	C3—H3B	0.9600
C8—C10	1.469 (3)	C3—H3C	0.9600
C13—C14	1.367 (3)	C1—H1A	0.9600
C13—C12	1.464 (3)	C1—H1B	0.9600
C5—C4	1.393 (3)	C1—H1C	0.9600
C5—C6	1.394 (4)	C2—H2A	0.9600
C4—C9	1.389 (3)	C2—H2B	0.9600
C10—C11	1.317 (3)	C2—H2C	0.9600
			
C16—S1—C13	91.44 (11)	O2—C6—C7	124.4 (2)
C5—O3—C2	115.8 (2)	O2—C6—C5	115.6 (2)
C6—O2—C1	117.5 (2)	C7—C6—C5	120.0 (2)
C4—O4—C3	118.39 (19)	C4—C9—C8	119.9 (2)
C7—C8—C9	119.9 (2)	C4—C9—H9	120.1
C7—C8—C10	121.1 (2)	C8—C9—H9	120.1
C9—C8—C10	118.9 (2)	C10—C11—C12	123.3 (2)
C14—C13—C12	130.7 (2)	C10—C11—H11	118.4
C14—C13—S1	110.55 (16)	C12—C11—H11	118.4
C12—C13—S1	118.78 (16)	O1—C12—C13	120.2 (2)
O3—C5—C4	120.6 (2)	O1—C12—C11	123.2 (2)
O3—C5—C6	119.6 (2)	C13—C12—C11	116.6 (2)
C4—C5—C6	119.7 (2)	O4—C3—H3A	109.5
O4—C4—C9	124.6 (2)	O4—C3—H3B	109.5
O4—C4—C5	115.3 (2)	H3A—C3—H3B	109.5
C9—C4—C5	120.2 (2)	O4—C3—H3C	109.5
C11—C10—C8	126.7 (2)	H3A—C3—H3C	109.5
C11—C10—H10	116.6	H3B—C3—H3C	109.5
C8—C10—H10	116.6	O2—C1—H1A	109.5
C6—C7—C8	120.2 (2)	O2—C1—H1B	109.5
C6—C7—H7	119.9	H1A—C1—H1B	109.5
C8—C7—H7	119.9	O2—C1—H1C	109.5
C16—C15—C14	111.8 (2)	H1A—C1—H1C	109.5
C16—C15—H15	124.1	H1B—C1—H1C	109.5
C14—C15—H15	124.1	O3—C2—H2A	109.5
C15—C16—S1	112.61 (17)	O3—C2—H2B	109.5
C15—C16—I1	128.15 (17)	H2A—C2—H2B	109.5
S1—C16—I1	119.23 (12)	O3—C2—H2C	109.5
C13—C14—C15	113.6 (2)	H2A—C2—H2C	109.5
C13—C14—H14	123.2	H2B—C2—H2C	109.5
C15—C14—H14	123.2		
			
C16—S1—C13—C14	0.39 (18)	C16—C15—C14—C13	0.0 (3)
C16—S1—C13—C12	179.91 (19)	C1—O2—C6—C7	3.0 (4)
C2—O3—C5—C4	84.7 (3)	C1—O2—C6—C5	−176.2 (3)
C2—O3—C5—C6	−99.0 (3)	C8—C7—C6—O2	−179.5 (2)
C3—O4—C4—C9	1.2 (4)	C8—C7—C6—C5	−0.3 (4)
C3—O4—C4—C5	−179.1 (2)	O3—C5—C6—O2	5.0 (3)
O3—C5—C4—O4	−6.1 (3)	C4—C5—C6—O2	−178.7 (2)
C6—C5—C4—O4	177.6 (2)	O3—C5—C6—C7	−174.3 (2)
O3—C5—C4—C9	173.6 (2)	C4—C5—C6—C7	2.0 (4)
C6—C5—C4—C9	−2.7 (4)	O4—C4—C9—C8	−178.7 (2)
C7—C8—C10—C11	11.7 (4)	C5—C4—C9—C8	1.6 (4)
C9—C8—C10—C11	−169.6 (3)	C7—C8—C9—C4	0.2 (4)
C9—C8—C7—C6	−0.8 (4)	C10—C8—C9—C4	−178.6 (2)
C10—C8—C7—C6	177.9 (2)	C8—C10—C11—C12	−177.4 (2)
C14—C15—C16—S1	0.3 (3)	C14—C13—C12—O1	177.4 (2)
C14—C15—C16—I1	179.10 (17)	S1—C13—C12—O1	−2.1 (3)
C13—S1—C16—C15	−0.38 (19)	C14—C13—C12—C11	−2.2 (4)
C13—S1—C16—I1	−179.33 (13)	S1—C13—C12—C11	178.35 (17)
C12—C13—C14—C15	−179.8 (2)	C10—C11—C12—O1	0.1 (4)
S1—C13—C14—C15	−0.3 (3)	C10—C11—C12—C13	179.6 (2)

### Hydrogen-bond geometry (Å, º)

**Table:**

*D*—H···*A*	*D*—H	H···*A*	*D*···*A*	*D*—H···*A*
C15—H15···O2^i^	0.93	2.45	3.341 (3)	159

Symmetry code: (i) *x*+1/2, *y*, −*z*+1/2.
